# Supplementary material for: The causal relationship between the human gut microbiota and pyogenic arthritis: a Mendelian randomization study
Source: Front Cell Infect Microbiol. 2024 Nov 26;14:1452480. doi: 10.3389/fcimb.2024.1452480 (PMC11629706; doi:10.3389/fcimb.2024.1452480)
Supplement: Supplementary file 1 [file DataSheet1.zip › mendelian test/ebi-a-GCST90027625.csv_leave_one_out.pdf]

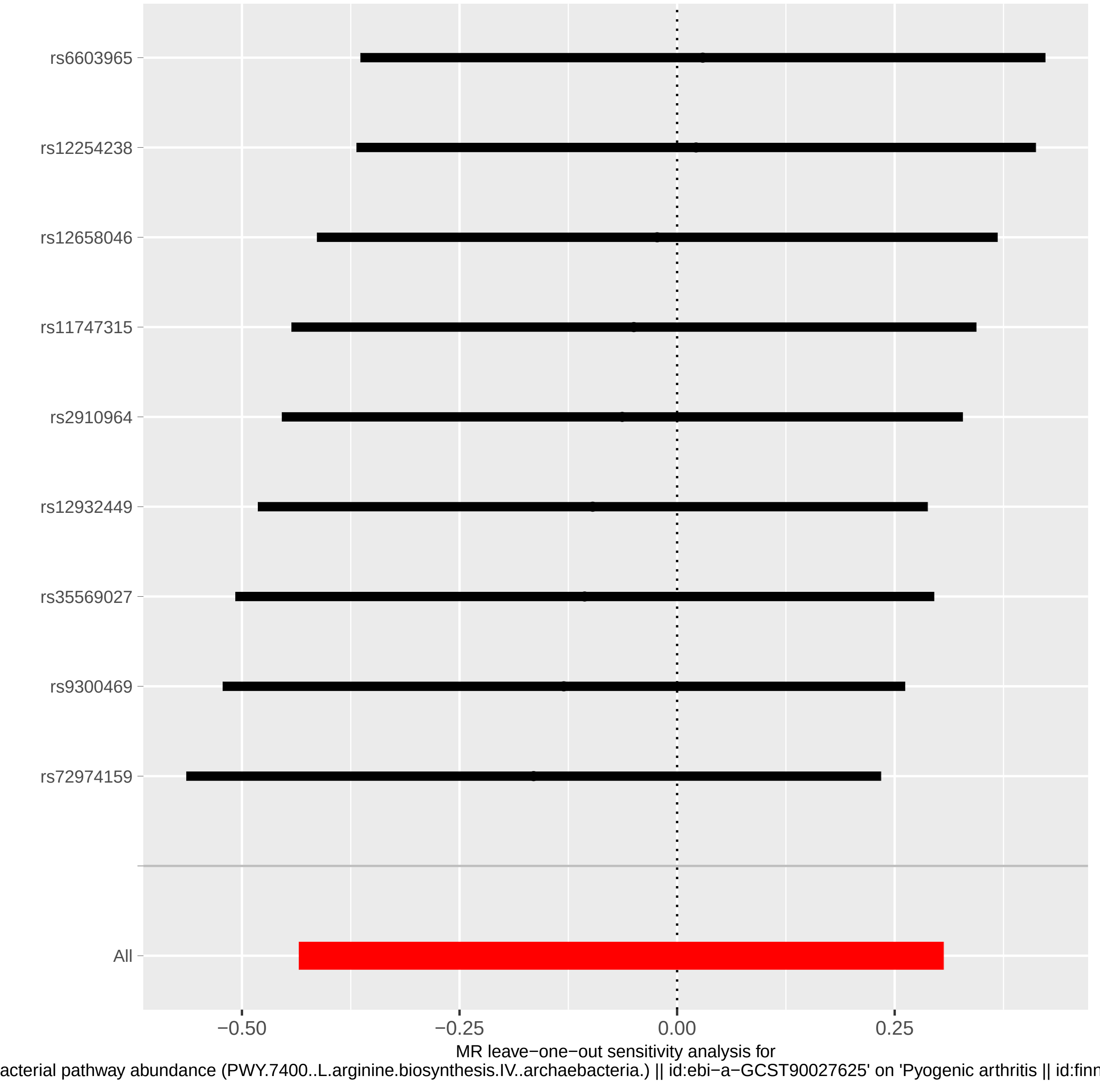
rs6603965
rs12254238
rs12658046
rs11747315
rs2910964
rs12932449
rs35569027
rs9300469
rs72974159
All
−0.50
−0.25
0.00
0.25
MR leave−one−out sensitivity analysis for
acterial pathway abundance (PWY.7400..L.arginine.biosynthesis.IV..archaebacteria.) || id:ebi−a−GCST90027625' on 'Pyogenic arthritis || id:finn
